# 2131. Activity of the Novel Antibiotic Zosurabalpin (RG6006) against Clinical *Acinetobacter* Isolates from China

**DOI:** 10.1093/ofid/ofad500.1754

**Published:** 2023-11-27

**Authors:** Stephen Hawser, Nimmi Kothari, Thomas Valmont, Séverine Louvel, Claudia Zampaloni

**Affiliations:** IHMA Europe, Monthey, Valais, Switzerland; IHMA, Monthey, Valais, Switzerland; IHMA, Monthey, Valais, Switzerland; F. Hoffmann-La Roche Ltd, Basel, Basel-Stadt, Switzerland; F. Hoffmann-La Roche Ltd., Immunology, Infectious Disease and Ophthalmology, Roche Innovation Center Basel, F. Hoffmann-La Roche Ltd, Grenzacherstrasse 124, 4070 Basel, Switzerland, Basel, Basel-Stadt, Switzerland

## Abstract

**Background:**

Zosurabalpin (RG6006) is the first representative of a novel class of tethered macrocyclic peptide antibiotics active against *Acinetobacter* spp., including carbapenem-resistant *Acinetobacter baumannii-calcoaceticus* complex (ABC) organisms. In this study, the susceptibility testing of zosurabalpin was carried out against a panel of 150 randomly selected *Acinetobacter* spp. isolates (100 *A. baumannii* & 50 non-*A. baumannii*) representing 11 sites in China and a broad susceptibility profile (65% of which were multi-drug resistant [MDR]) collected in 2021.

**Methods:**

MICs were performed using the Clinical Laboratory Standards Institute (CLSI) broth dilution method cation-adjusted Mueller Hinton broth (CA-MHB) supplemented with either 20% of human serum (HS) or 20% of horse serum (HoS). For a fraction of isolates (10-25%), MIC determination for zosurabalpin is affected by aberrant readings (trailing, multiple skipped wells) in CA-MHB. This effect complicates routine susceptibility testing. Supplementation of CAMHB with serum allows accurate MIC determinations without any major effects on the MIC distribution.

**Results:**

Summary data from the study are shown in Table 1.

Zosurabalpin was active against all *Acinetobacter* spp., with an MIC_50/90_ of 0.12/0.5 μg/mL and 0.25/1 μg/mL in CA-MHB supplemented with HoS and HS, respectively (MIC range of 0.015/0.03 to 8 μg/mL). Against ABC isolates (n=133), the MIC_50/90_ for zosurabalpin was 0.12/0.25 μg/mL and 0.25/0.5 μg/mL, in HoS and HS, respectively (MIC range of 0.015/0.03 to 1 μg/mL). A similar activity was observed against carbapenem-resistant ABC isolates.

**Figure:**
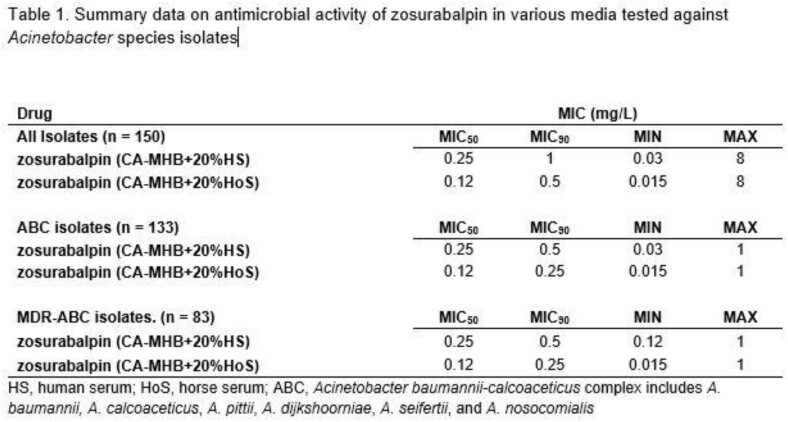

**Conclusion:**

In addition to the potent activity observed against isolates from USA and Europe, zosurabalpin exhibited potent *in vitro* antibacterial activity against *Acinetobacter* clinical isolates circulating in China. These data support the continued clinical development of zosurabalpin for infections caused by ABC isolates, including difficult to treat carbapenem-resistant isolates.

**Disclosures:**

**Stephen Hawser, PhD**, Allecra: study funding|Innoviva Specialty Therapeutics, Inc.: Honoraria|Roche: Honoraria|Roche: This project has been funded by BARDA (HHSO100201600038C). **Nimmi Kothari, PhD**, Allecra: Allecra (study funding)|Innoviva Specialty Therapeutics, Inc.: Honoraria|Roche: Honoraria|Roche: This project has been funded by BARDA (HHSO100201600038C). **Thomas Valmont, BS**, Roche: Honoraria|Roche: This project has been funded by BARDA (HHSO100201600038C). **Séverine Louvel, PhD**, F. Hoffmann-La Roche Ltd: employee of the company **Claudia Zampaloni, n/a**, F. Hoffmann-La Roche Ltd.: Full time employee of Roche

